# Supplementary material for: Mammal communities are larger and more diverse in moderately developed areas
Source: eLife. 2018 Oct 2;7:e38012. doi: 10.7554/eLife.38012 (PMC6168282; doi:10.7554/eLife.38012)
Supplement: Supplementary file 1. [file elife-38012-supp1.docx]

| Supplementary file 1: Effort expressed as camera nights with spatial replicates in parentheses for camera traps run in Washington, DC and Raleigh, NC from 2012-2016 between different levels along the urban-wild gradient around each city. | | | | | |
| --- | --- | --- | --- | --- | --- |
| Washington, DC | | | | | |
| Gradient Level | Large Forest >10 km^2^ | Small Forest $\leq$10 km^2^ | Yard | Open | Total |
| Urban | 0 (0) | 430.3 (12) | 0 (0) | 0 (0) | 430.3 (12) |
| Suburban | 517.4 (25) | 530 (21) | 109.4 (5) | 0 (0) | 1156.8 (51) |
| Exurban | 671.8 (31) | 150.5 (7) | 281 (14) | 0 (0) | 1103.3 (52) |
| Rural | 737.4 (35) | 1699 (80) | 598.3 (27) | 0 (0) | 3034.7 (142) |
| Wild | 4076 (176) | 1720.6 (79) | 0 (0) | 0 (0) | 5796.6 (255) |
| Total | 6002.6 (267) | 4530.3 (199) | 988.8 (46) | 0 (0) | 11521.6 (512) |
| Raleigh, NC | | | | | |
| Suburban | 105.9 (4) | 3346 (152) | 2981.9 (149) | 1070.3 (55) | 7504.2 (360) |
| Exurban | 251.5 (12) | 2673.6 (120) | 1545.9 (77) | 956.1 (49) | 5427.1 (258) |
| Rural | 603.2 (27) | 1801.4 (83) | 637.1 (27) | 1059.9 (51) | 4101.5 (188) |
| Wild | 987.7 (43) | 1322.7 (66) | 0 (0) | 0 (0) | 2310.3 (109) |
| Total | 1948.4 (86) | 9143.6 (421) | 5164.9 (253) | 3086.3 (155) | 19343.1 (915) |
