# Supplementary material for: Mammal communities are larger and more diverse in moderately developed areas
Source: eLife. 2018 Oct 2;7:e38012. doi: 10.7554/eLife.38012 (PMC6168282; doi:10.7554/eLife.38012)
Supplement: Supplementary file 2. [file elife-38012-supp2.docx]

| Supplementary file 2. Covariates used in the detection rate and occupancy analyses. | | | |
| --- | --- | --- | --- |
| Covariate | Description | Type | Model |
| City | 0/1 Washington, DC or Raleigh, NC. Interactions between this term and all other covariates were tested. | Camera site | Ψ |
| Large Forest | % Large core (cont. forest frag >5 acre) in 5 km radius | GIS 5 km | Ψ |
| Small Tree Cover | % tree cover in 100 m radius (Sexton et al., 2013) | GIS 100 m |  |
| Housing Density | Average Housing Density (houses/km2) in 5km radius (Hammer et al., 2004) | GIS 5 km | Ψ |
| Large Forest x Housing Density | Interaction of large core forest and housing density in a 5 km radius | Interaction | Ψ |
| Small Tree Cover x Housing Density | Interaction of small scale (100 m) tree cover and housing density in a 5 km radius | Interaction | Ψ |
| Rolag Rate | Site-specific detection rate (count/day) of rodents and lagomorphs | Camera site | Ψ |
| Deer Rate | Site-specific detection rate (count/day) of white-tailed deer | Camera site | Ψ |
| Hunting | Categorical covariate for hunting or no hunting is permitted at the site (0,1) | Camera site | Ψ |
| Year | Year sampled | Camera site | Ψ |
| Dog | 0/1 variable representing if a dog is located at the site (yards only) | Camera site | Ψ |
| Dog x Yard | An interaction term between dog presence and whether or not a site is a yard | Interaction | Ψ |
| Yard | 0/1 variable representing if the camera was located inside or outside of a residential yard | Camera site | Ψ, p |
| NDVI | Moderate Resolution Imaging Spectroradiometer Land Terra Vegetation Indices 1 km monthly NDVI (Dodge et al., 2013) | Camera site/Day | p |
| Temperature | ECMWF Interim Full Daily SFC Temp (2m above ground) (Dodge et al., 2013) | Camera site/Day | p |
| Camera (Raleigh) | 0/1 variable representing the type of camera used (0=Bushnell, 1=Reconyx) | Camera site | p |
| Detection distance | Maximum distance at which camera detects animals (m) | Camera site | p |
