# Supplementary material for: Mammal communities are larger and more diverse in moderately developed areas
Source: eLife. 2018 Oct 2;7:e38012. doi: 10.7554/eLife.38012 (PMC6168282; doi:10.7554/eLife.38012)
Supplement: Supplementary file 3. [file elife-38012-supp3.docx]

| Supplementary file 3: Results of goodness-of-fit tests for occupancy and Poisson count models assessed by posterior predictive check with adequate fit if 0.1 < pB < 0.9. | | |
| --- | --- | --- |
| Species | Occupancy | Count Model |
| Bobcat | 0.32 | 0.28 |
| Coyote | 0.24 | 0.5 |
| Gray Fox | 0.26 | 0.34 |
| Red Fox | 0.30 | 0.5 |
