# Supplementary material for: Mammal communities are larger and more diverse in moderately developed areas
Source: eLife. 2018 Oct 2;7:e38012. doi: 10.7554/eLife.38012 (PMC6168282; doi:10.7554/eLife.38012)
Supplement: Supplementary file 4. [file elife-38012-supp4.docx]

Supplementary File 4: Calculating goodness of fit statistics

- 1. **Pearson residuals**

The squared Pearson residual is defined as:

$$r_{P}^{2}=\frac{{(y-\mu)}^{2}}{V(\mu)}$$

where $y$ is an observed response variable, $E\left( Y \right)=\mu$, and $V(\mu)$ is the variance function (McCullagh and Nelder 1989, equation 2.11).

**1.1.1 Occupancy Models**

Occupancy models (sensu MacKenzie et al. 2017) are defined by the following joint distribution of $z$, the partially observed latent state, and $y$, detection / non-detections of the underlying state:

$$z\sim Bernoulli\left( \psi\right)$$

$$y\sim Bernoulli(zp)$$

This can alternatively be written as:

$$f\left( z,y \right)=\psi^{z}{(1-\psi)}^{1-z}({zp)}^{y}{(1-zp)}^{1-y}$$

Since we only fully observe $y$, we marginalize over $z$ to obtain the distribution of $y$:

$$f\left( y \right)={\psi p}^{y}{(1-p)}^{1-y}+(1-\psi)0^{y}$$

To calculate squared Pearson's residuals, we first obtain $E\left( y \right)$ as:

$$E\left( Y \right)=\sum_{y} yf(y)$$

$$=\psi p$$

To obtain $V(\mu)$, we first obtain the variance of $y$ as:

$$Var\left( Y \right)={E(Y}^{2})-{E(Y)}^{2}$$

$$=\sum_{y} y^{2}f\left( y \right)-({\psi p)}^{2}$$

$$=\psi p-{(\psi p)}^{2}$$

$$=\psi p(1-\psi p)$$

Since $E\left( Y \right)=\mu=\psi p, V\left( \mu\right)=\mu\left( 1-\mu\right)=\psi p(1-\psi p)$ we can now write a squared Pearson's residual as:

$$r_{P}^{2}=\frac{(y-{\psi p)}^{2}}{\psi p(1-\psi p)}$$

**Literature Cited**

MacKenzie, D., J. Nichols, J. Royle, K. Pollock, L. Bailey, and J. Hines. 2017. Occupancy Estimation and Modeling: Inferring Patterns and Dynamics of Species Occurrence, 2^nd^ Edition. Academic Press, London, UK.

McCullagh, P., and J.A. Nelder. 1989. Generalized Linear Models, Second Edition. Chapman & Hall, Boca Raton, Florida, USA.
