# Supplementary material for: Mammal communities are larger and more diverse in moderately developed areas
Source: eLife. 2018 Oct 2;7:e38012. doi: 10.7554/eLife.38012 (PMC6168282; doi:10.7554/eLife.38012)
Supplement: Supplementary file 5. — Also presented are results of a separate Poisson regression to determine differences in detection rate between yards and all other plot types. Significant results (95% CIs not overlapping zero) are in bold. [file elife-38012-supp5.docx]

| Supplementary file 5: Results of a Poisson regression to determine differences in detection rate between the wild gradient level (reference level) and all other levels of the development gradient (above dotted line). Also presented are results of a separate Poisson regression to determine differences in detection rate between yards and all other plot types. Significant results (95% CIs not overlapping zero) are in bold. | | | | |
| --- | --- | --- | --- | --- |
| Washington, DC | | | | |
| Predictor | Mean | SD | 2.5% | 97.5% |
| Intercept | -0.07 | 0.06 | -0.18 | 0.06 |
| Rural | **0.25** | 0.1 | 0.06 | 0.45 |
| Exurban | **0.51** | 0.13 | 0.25 | 0.77 |
| Suburban | **0.69** | 0.13 | 0.44 | 0.96 |
| Urban | **0.72** | 0.27 | 0.16 | 1.22 |
| Intercept | **0.54** | 0.15 | 0.26 | 0.83 |
| Large Forest | **-0.46** | 0.17 | -0.77 | -0.13 |
| Small Forest | **-0.42** | 0.17 | -0.77 | -0.11 |
| Raleigh, NC | | | | |
| Intercept | -0.51 | 0.13 | -0.77 | -0.28 |
| Rural | 0.15 | 0.17 | -0.18 | 0.46 |
| Exurban | **0.32** | 0.16 | 0.02 | 0.67 |
| Suburban | 0.07 | 0.15 | -0.21 | 0.34 |
| Intercept | **-0.37** | 0.09 | -0.56 | -0.2 |
| Large Forest | 0.05 | 0.17 | -0.3 | 0.35 |
| Small Forest | 0.15 | 0.12 | -0.09 | 0.38 |
| Open | **-0.42** | 0.15 | -0.68 | -0.13 |
