# Supplementary material for: Mammal communities are larger and more diverse in moderately developed areas
Source: eLife. 2018 Oct 2;7:e38012. doi: 10.7554/eLife.38012 (PMC6168282; doi:10.7554/eLife.38012)
Supplement: Supplementary file 6. — Posterior mean and posterior standard deviation for each predictor are shown with bold entries indicating predictors with 95% credible intervals that did not overlap zero. Predictors in bold were used for modeling occupancy for that species. Housing density was used as a predictor in all occupancy models, regardless of whether it was significant in the preliminary count analysis. [file elife-38012-supp6.docx]

| Supplementary file 6: Results of Poisson regression for single species using camera trapping over two cities between 2012-2016. Posterior mean and posterior standard deviation for each predictor are shown with bold entries indicating predictors with 95% credible intervals that did not overlap zero. Predictors in bold were used for modeling occupancy for that species. Housing density was used as a predictor in all occupancy models, regardless of whether it was significant in the preliminary count analysis. | | | | |
| --- | --- | --- | --- | --- |
| Covariate | Bobcat | Coyote | Gray Fox | Red Fox |
| Intercept | **-12.31(2.14)** | **-7.61(0.88)** | **-10.08(1.63)** | **-4.14(0.64)** |
| Hunting | -1.09(0.83) | **1.43(0.4)** | -1.54(1.31) | 0.35(0.33) |
| Yard | -3.03(3.92) | 0.24(1.09) | -2.8(3.2) | **1.54(0.65)** |
| Dog | 3.45(4.11) | -0.37(1.44) | **7.71(3.27)** | 0.32(1.18) |
| Year | -0.85(0.53) | **-0.57(0.24)** | 0.71(0.75) | 0.26(0.19) |
| Forest | 1.34(1.13) | **0.88(0.33)** | 1.27(0.92) | **-0.68(0.29)** |
| Housing Density | -2.85(2.93) | **1.26(0.53)** | **1.69(0.77)** | 0.6(0.49) |
| Large Forest x Housing Density | 1.32(1.26) | **0.81(0.31)** | **0.97(0.35)** | 0.39(0.29) |
| Rolag Rate | -2.64(4.13) | -1.99(2.69) | -1.23(5.16) | 3.16(1.95) |
| Deer Rate | -2.17(1.92) | **-2.3(1.47)** | -2.47(1.51) | **-2.25(1.2)** |
| Small Tree Cover | -0.05(1.12) | -0.06(0.37) | -0.68(0.69) | -0.09(0.26) |
| Housing Density x Small Tree Cover | 1.09(1.33) | 0.37(0.22) | 0.12(0.22) | -0.1(0.14) |
| Yard*Dog | 1.04(2.07) | 0.66(0.71) | -0.28(0.69) | -1.21(0.87) |
| City | 2.27(1.76) | **2.23(0.86)** | **4.27(1.62)** | **-3.9(0.7)** |
| City x Hunting | **2.78(1.16)** | -0.45(0.49) | 1.9(1.39) | -0.66(0.67) |
| City x Yard | 1.82(3.89) | -1.37(1.16) | 2.88(3.22) | -0.19(0.79) |
| City x Dog | -3.3(4.09) | 0.24(1.39) | **-7.24(3.24)** | 0.94(1) |
| City x Year | 0.77(0.68) | **0.64(0.28)** | -1.48(0.77) | -0.03(0.3) |
| City x Large Forest | **-2.67(1.02)** | -0.56(0.38) | -0.71(0.96) | **1.12(0.54)** |
| City x Housing Density | -0.42(2.61) | -0.95(0.57) | 0.02(0.78) | 0.06(0.75) |
| City x Rolag Rate | 2.61(4.15) | 2.13(2.69) | 1.4(5.16) | -3.06(1.95) |
| City x Deer Rate | 5.34(5.48) | **11.63(2.59)** | **9.22(3.21)** | 1.3(4.9) |
| City x Small Tree Cover | 0.68(0.83) | 0.16(0.38) | 0.38(0.71) | 0.11(0.33) |
