# Supplementary material for: Mammal communities are larger and more diverse in moderately developed areas
Source: eLife. 2018 Oct 2;7:e38012. doi: 10.7554/eLife.38012 (PMC6168282; doi:10.7554/eLife.38012)
Supplement: Supplementary file 7. — Those coefficients with 95% CIs not overlapping zero are shown in bold. [file elife-38012-supp7.docx]

| Supplementary file 7: Beta coefficients and 95% credible intervals (parentheses) for an occupancy model based on camera trapping data in Washington, DC, USA and Raleigh, NC, USA from 2012-2016. Those coefficients with 95% CIs not overlapping zero are shown in bold. | | | | |
| --- | --- | --- | --- | --- |
|  | Bobcat | Coyote | Gray Fox | Red Fox |
| Intercept | 1.1 (-1.5, 4.5) | -1.3 (-2.8, 0.1) | -1.2 (-2.7, 0.2) | -1.3 (-2.8, 0.2) |
| Housing Density | -1.8 (-5.4, 1.4) | -0.1 (-1, 0.8) | **1 (0.2, 1.8)** | 0.2 (-0.7, 1) |
| Large Forest | 0.6 (-1.7, 3.3) | 0.3 (-0.2, 0.9) | 0.4 (-0.1, 0.9) | 0.5 (0, 1) |
| Housing Density x Large Forest | -0.4 (-3.4, 1.9) | -0.1 (-0.6, 0.4) | **0.6 (0.1, 1)** | 0.1 (-0.3, 0.6) |
| Hunting | 0.4 (-2.3, 3.7) | 0.1 (-0.5, 0.7) | -0.4 (-1.1, 0.2) | **0.6 (0.1, 1.2)** |
| Yard | 0.7 (-2.4, 4.1) | -0.7 (-2.2, 0.8) | 0.2 (-1.3, 1.6) | 0.4 (-1.1, 1.8) |
| Dog | -0.8 (-3.9, 2) | -0.8 (-3, 1.2) | 0.3 (-1.6, 2.2) | -0.1 (-2, 1.9) |
| Rolag Rate | -0.2 (-3.8, 3.5) | -0.9 (-4, 1.9) | -0.4 (-2.7, 2.3) | -1.9 (-5.9, 0.7) |
| Deer Rate | -0.7 (-2.9, 1.5) | -0.2 (-0.6, 0.1) | -0.1 (-0.4, 0.3) | 0 (-0.4, 0.3) |
| Year | 0.2 (-3.4, 3.8) | 0.5 (-0.2, 1.3) | 0.4 (-0.1, 0.9) | 0.2 (-0.3, 0.8) |
| Dog*Yard | 0.5 (-2.8, 4.2) | 0.5 (-0.7, 1.7) | -0.7 (-1.6, 0.3) | 0.3 (-1, 1.6) |
| Small Tree Cover | -1.4 (-4.5, 1.1) | 0.2 (-0.3, 0.8) | 0.4 (-0.2, 1.1) | -0.1 (-0.6, 0.4) |
| Housing Density x Small Tree Cover | 0 (-2.9, 2.7) | 0.1 (-0.2, 0.4) | -0.2 (-0.4, 0.1) | 0.1 (-0.1, 0.4) |
| City | 0.9 (-1.7, 3.9) | 0.4 (-0.4, 1.3) | 0.6 (-0.2, 1.4) | -0.4 (-1.2, 0.4) |
| City x Large Forest | -0.1 (-2.9, 2.5) | 0.1 (-0.6, 0.8) | 0.2 (-0.5, 0.8) | -0.7 (-1.3, 0) |
| City x Hunting | -0.8 (-4.4, 2.7) | -0.1 (-1, 0.8) | 0.4 (-0.5, 1.2) | 0 (-0.9, 0.9) |
| City x Year | 0.1 (-2.9, 3.8) | 0.3 (-0.2, 0.8) | -0.1 (-0.6, 0.3) | 0.1 (-0.4, 0.5) |
| City x Deer Rate | 0 (-3.7, 3.5) | -0.3 (-3.6, 2.9) | 1 (-1.8, 4.1) | -1.2 (-5.1, 2) |
| City x Dog | 0.2 (-3.1, 3.8) | -0.1 (-1.5, 1.3) | 0.1 (-1.2, 1.5) | **-1.4 (-2.7, -0.2)** |
| City x Housing Density | 0.6 (-2.5, 3.9) | 0.7 (-0.3, 1.7) | 0.4 (-0.5, 1.3) | -0.5 (-1.4, 0.4) |
| City x Yard | -0.8 (-3.8, 2.2) | 0.8 (-1.2, 3) | -0.3 (-2.2, 1.5) | 0 (-2, 1.9) |
| City x Rolag Rate | -1.1 (-4.5, 1.7) | 0 (-0.6, 0.6) | -0.1 (-0.8, 0.6) | 0 (-0.5, 0.5) |
| City x Small Tree Cover | -0.1 (-3.8, 3.6) | 0 (-1.9, 2) | 1.2 (-0.7, 3.2) | -0.2 (-2.5, 1.8) |
